# Outcomes of Outpatient Advanced Therapy Exposed Patients Hospitalized With Severe Ulcerative Colitis

**DOI:** 10.1093/crocol/otaf055

**Published:** 2025-08-12

**Authors:** Badr Al-Bawardy, Eman Al Sulais, Fatimah AlHarthi, Gamal Mohamed, Mariam S Mukhtar, Ailsa Hart, Tim Raine

**Affiliations:** Division of Gastroenterology and Hepatology, Department of Internal Medicine, King Faisal Specialist Hospital, Riyadh, Saudi Arabia; College of Medicine, Alfaisal University, Riyadh, Saudi Arabia; Department of Internal Medicine, Section of Digestive Diseases, Yale School of Medicine, New Haven, CT, United States; Department of Medicine, King Fahad Specialist Hospital, Dammam, Saudi Arabia; Department of Biostatistics, Epidemiology and Scientific Computing, King Faisal Specialist Hospital, Riyadh, Saudi Arabia; Department of Biostatistics, Epidemiology and Scientific Computing, King Faisal Specialist Hospital, Riyadh, Saudi Arabia; Department of Internal Medicine, Faculty of Medicine, King Abdulaziz University, Jeddah, Saudi Arabia; IBD Unit, St Mark’s Hospital, London, United Kingdom; Department of Gastroenterology, Addenbrooke’s Hospital, Cambridge University Hospitals NHS Foundation Trust, Cambridge, United Kingdom

**Keywords:** ulcerative colitis, acute severe ulcerative colitis, biologic, small molecules

## Abstract

**Background:**

Contemporary characteristics of hospitalized patients with ulcerative colitis (UC) may differ from historic standards in terms of prior drug exposure and disease severity. The impact of these differences on outcomes is unclear. This study aimed to assess inpatient UC outcomes according to prior outpatient drug exposure and measures of disease severity.

**Methods:**

This was a multicenter, retrospective study of adult patients (age ≥ 18 years) hospitalized for severe UC. The primary outcome was the colectomy rate among outpatient advanced therapy exposed (ATE) vs advanced therapy naïve (ATN) patients. Secondary outcomes included length of hospitalization and need for rescue medical therapy.

**Results:**

A total of 370 patients were included with 86 (23%) in the ATE group and 284 (77%) in the ATN group. In the ATE group, 21 patients (25%) required colectomy vs 26 (9%) in the ATN group (*P* < .001). Median hospital length of stay was 6 days (IQR: 4-9) in both groups (*P* = .96). Rescue medical therapy was required in 107 (38%) patients in the ATN group vs 36 (42%) in the ATE group (*P* = .49).

Colectomy was associated with ATE status (*P* = .0002), Mayo UC endoscopic sub-score of 3 (*P* = .002), higher C-reactive protein (*P* = .04), lower albumin (*P* = .0002), and female sex (*P* = .03). On multivariable analysis, only low albumin was independently associated with colectomy (*P* = .001).

**Conclusions:**

Outpatient ATE was associated with an increased risk of colectomy among hospitalized patients with severe UC. On multivariable analysis, low albumin was independently associated with the risk of colectomy. This suggests that higher colectomy rates observed in ATE patients may reflect underlying differences in disease severity.

## Introduction

Ulcerative colitis (UC) is a chronic inflammatory bowel disease characterized by relapsing symptoms of diarrhea, rectal bleeding, and abdominal pain. Despite advancements in diagnosis, monitoring, and therapies to treat UC, a significant proportion of patients will develop a severe flare requiring hospitalization. The prevalence of an acute severe flare in patients with UC has been estimated to be around 20%-30%.^1^^,^^2^ In a more recent systematic review and meta-analysis, the 5-year risk of hospitalizations in UC was found to be 21.5%.^3^ A global temporal study demonstrated stability in the rates of hospitalizations due to UC in the Western world, but a continued increase in the rate of UC hospitalizations in newly industrialized countries.^4^

Acute severe ulcerative colitis (ASUC) may be defined according to the Truelove and Witts criteria, requiring the occurrence of bloody stool frequency > 6/day; and one of the following as evidence of systemic toxicity: temperature >37.8 °C, pulse >90 beats/min, hemoglobin <10.5 g/dL, erythrocyte sedimentation >30 mm/hr.^5^ These criteria may also reflect the risk of colectomy in such patients. For example, in a retrospective case series, the risk of colectomy for patients admitted with ASUC ranged from 8.5% if 1 criterion was met to 48% if 3 or more criteria were met.^1^ A notable limitation is that the Truelove and Witts criteria, as with other scores for ASUC such as the Lichtiger score and Oxford criteria, were developed prior to the advent of advanced therapies.^6^^,^^7^ Therefore, it is unclear if these criteria are optimal to assess disease severity and predict disease outcomes in patients who have already been exposed to advanced therapy in the outpatient setting.

In hospitalized UC patients previously exposed to immunomodulators, limited studies have suggested an association between outpatient immunomodulator exposure and unfavorable outcomes such as lack of response to corticosteroids and short-term colectomy.^8^^,^^9^ Previous exposure to biologic therapy has been reported to be associated with lower response rates to subsequent therapies, as noted in multiple clinical trials.^10–13^ Data regarding outcomes of hospitalized severe UC patients have mainly included or focused on patients who are naïve to advanced therapies. Even in more recent cohorts of ASUC, the majority of the population studied are advanced therapy naïve (ATN), and outcomes of advanced therapy exposed (ATE) patients have not been reported separately.^14^ However, a few recent studies have suggested that previous anti-tumor necrosis factor (TNF) exposure in the outpatient setting may be associated with increased risk of colectomy in ASUC.^14,15^ It is not clear if any increased risk of colectomy reflects more aggressive disease in this patient population, or alterations in clinician decision-making in a population who may be perceived as more refractory. Therefore, we aimed to assess outcome data for patients hospitalized with severe UC according to prior outpatient exposure to advanced therapies and assess the impact of measures of disease severity.

## Methods

### Study design

We performed a multicenter, retrospective study of patients hospitalized with severe UC. We included data from 3 centers: 2 from the United Kingdom (Addenbrooke’s Hospital and St Mark’s Hospital) and 1 from the United States (Yale University). The study protocol was approved by the Yale University Institutional Review Board (IRB# 2000031140). In the Yale University cohort, we included all patients who were hospitalized for a severe UC flare from January 1, 2012 to November 1, 2021. The UK cohort included patients who were hospitalized for a severe UC flare from April 2015 to October 2022. We collected demographic information at the time of admission, including age, sex, body mass index, and smoking status, as well as disease-related variables, including date of UC diagnosis, disease location, and presence of documented extraintestinal manifestations. Other variables included all current and prior medical therapies for UC, dates and times of hospital admission, flexible sigmoidoscopy findings, presence of cytomegalovirus by histopathology, *Clostridioides difficile* (*C. difficile*) infection, labs including admission C-reactive protein (CRP) and albumin, and need for rescue medical therapy or colectomy during the admission.

### Patients

We included data from adult patients (age ≥ 18 years) with a confirmed diagnosis of UC, and hospitalization due to a UC flare requiring treatment with intravenous corticosteroids. We excluded pediatric patients (age < 18 years), or a diagnosis of Crohn’s disease or inflammatory bowel disease (IBD)-unclassified. The outcomes of patients exposed to advanced therapies in the outpatient setting were compared to those who were naïve to advanced therapies. Patients were considered exposed to advanced therapies if they had received at least 1 dose of the following medications: infliximab, adalimumab, golimumab, vedolizumab, ustekinumab, and tofacitinib.

### Outcomes

The primary outcome was the rate of colectomy compared between the ATE and ATN groups. Secondary outcomes included the rates of medical rescue therapy and the length of hospitalization. Need for rescue medical therapy was defined as requiring at least 1 dose of infliximab, cyclosporine, or tofacitinib in the inpatient setting to treat the UC flare.

### Statistical analysis

Descriptive statistics (numbers, percentages) were utilized for categorical variables, while continuous variables were summarized using mean with standard deviation (SD) or median with interquartile ranges (IQRs). The Chi-square test and the Wilcoxon rank sum test were used to examine the differences in characteristics between groups. Univariate analysis was conducted to explore the predictors of outcomes of interest, including the rate of colectomy and need for medical rescue therapy. Multivariate analysis was performed to assess the relationship between multiple predictors to the outcomes of interest (colectomy and medical rescue therapy). In the multivariate model, we included factors that demonstrated a *P*-value ≤ 0.1 in the univariate analysis. Results were reported as odds ratios (ORs with 95% confidence intervals (CIs). Data analysis was performed using Stata version 18 (StataCorp). All tests were 2-tailed, and statistical significance was set at a *P*-value < 05.

## Ethical Considerations

The study was approved by the Institutional Review Boards (IRB) of each institution involved in the study.

## Results

### Baseline characteristics

A total of 382 patients were reviewed. The median age was 35 years (IQR: 25-53), and 53% were male. The median disease duration was 3 years (IQR: 1-9) (Table 1). The US cohort made up 44% (*n* = 168), while the UK cohort made up 56% (*n* = 214) of the population studied. The US cohort was more likely to have pancolitis (70% vs 48%; *P* < .001) and meet the Truelove and Witts criteria (95% vs 50%; *P* < .001) on admission. The US cohort was more likely to be exposed to advanced therapies (35.1% vs 12.6%; *P* < .001) and have a higher baseline CRP and lower baseline albumin (Table 1). A total of 12 patients had missing data regarding advanced therapy exposure status and were excluded from further analysis.

**Table 1.** Baseline characteristics stratified by cohort population.

Variable	All cohort (*n* = 382)	US (*n* = 168)	UK (*n* = 214)	*P*-value
**Age (years), median (IQR)**	35 (25-53)	35 (26-53)	34 (24-50.3)	.47
Male, *n* **(%)**	202 (53.2)	88 (52.4)	114 (53.8)	.79
**Disease duration (years), median (IQR)**	3 (1-9)	2.5 (1-10)	3 (1-8)	.91
**Disease location,** *n* **(%)**				**<.001**
**Proctitis**	20 (5.7)	6 (3.6)	14 (7.7)	
**Left-sided colitis**	126 (36.0)	45 (26.8)	81 (44.5)	
**Pancolitis**	204 (58.3)	117 (69.6)	87 (47.8)	
**Smoking status,** *n* **(%)**				.22
**Current smoker**	14 (6.6)	9 (5.4)	5 (11.4)	
**Previous smoker**	63 (29.7)	48 (28.6)	15 (34.1)	
**Never smoker**	135 (63.7)	111 (66.1)	24 (54.6)	
**Extraintestinal manifestation,** *n* **(%)**	22 (8.9)	13 (7.7)	9 (11.7)	.32
**Advanced therapy exposed,** *n* **(%)**	86 (22.5)	59 (35.1)	27 (12.6)	**<.001**
**CRP (mg/dL), median (IQR)**	41.1 (10.8-96.3)	55.5 (19.6-119.3)	34 (9-85.2)	**.006**
**Albumin (g/dL), median (IQR)**	3.5 (2.9-3.9)	3.6 (3-3.9)	3.3 (2.8-3.9)	.07
**Anemia on admission,** *n* **(%)**	135 (35.7)	66 (39.3)	69 (32.9)	.19
**Truelove Witts criteria,** *n* **(%)**	261 (69.4)	158 (94.1)	103 (49.5)	**<.001**

Bold indicates *p*‑value < 0.05

Missing data (*n*): male (*n* = 2), disease duration (*n* = 29); disease location (*n* = 32), smoking status (*n* = 17), extraintestinal manifestation (*n* = 137), and advanced therapy naïve (*n* = 12).

Abbreviations: CRP, C-reactive protein; IQR, interquartile range; UK, United Kingdom; US: United States.

A total of 284 (77%) patients were naïve to advanced therapies, while 86 (23%) of patients were exposed to advanced therapies in the outpatient setting prior to the index hospitalization. Of those exposed to advanced therapies, 18 (21%) were exposed to 2 or more advanced therapies. A total of 52 patients were previously treated with infliximab, 25 were treated with adalimumab, 18 were treated with vedolizumab, 4 were treated with golimumab, 4 were treated with tofacitinib, and 2 were treated with ustekinumab.

The ATN group had a shorter median disease duration of 2 years (IQR: 1-8) vs 6 years (IQR: 2-11) in the ATE group (*P* < .001). The ATE group was more likely to have pancolitis compared to the ATN group (70% vs 55%; *P* = .04). The CRP to albumin ratio was significantly lower in the ATE group at 8 (IQR: 1-21.5) vs 12.8 (IQR: 3.2-35) (*P* = .004) (Table 2).

**Table 2.** Baseline characteristics stratified by advanced therapy exposure prior to hospitalization.

Variable	Advanced therapy naïve (*n* = 284)	Advanced therapy exposed (*n* = 86)	*P*-value
**Age (years), median (IQR)**	35 (25-53)	36.5 (24-53.3)	.86
**Male,** *n* **(%)**	152 (53.5)	43 (51.2)	.71
**Disease duration (years), median (IQR)**	2 (1-8)	6 (2-11)	**<.001**
**Disease location,** *n* **(%)**			**.04**
**Proctitis**	17 (6.4)	3 (3.6)	
**Left-sided colitis**	103 (38.9)	22 (26.2)	
**Pancolitis**	145 (54.7)	59 (70.2)	
**Smoking status,** *n* **(%)**			.53
**Current smoker**	11 (7.5)	3 (4.7)	
**Previous smoker**	46 (31.3)	17 (26.6)	
**Never smoker**	90 (61.22)	44 (68.8)	
**CRP (mg/dL) on admission, median (IQR)**	42.2 (13.9-99.7)	33 (9.5-75.8)	.19
**Albumin (g/dL) on admission, median (IQR)**	3.4 (2.8-3.9)	3.6 (3.1-3.9)	.08
**CRP/Albumin ratio, median (IQR)**	12.8 (3.2-35)	8 (1-21.5)	**.004**
**Anemia on admission,** *n* **(%)**	99 (35.1)	31 (36.1)	.87
**Mayo UC 3 endoscopic sub-score**	133 (59.9)	37 (57.8)	.90
**Truelove Witts Criteria,** *n* **(%)**	188 (67.4)	67 (77.9)	.06
**Concomitant CMV,** *n* **(%)**	13 (4.9)	6 (7.1)	.45
**Concomitant C. diff,** *n* **(%)**	3 (1.5)	2 (3.5)	.34

Bold indicates *p*‑value < 0.05

Abbreviations: C. diff, *Clostridioides difficile*; CMV, cytomegalovirus; CRP, C-reactive protein; IQR, interquartile range; UC, ulcerative colitis.

### Outcomes

**Figure 1.**
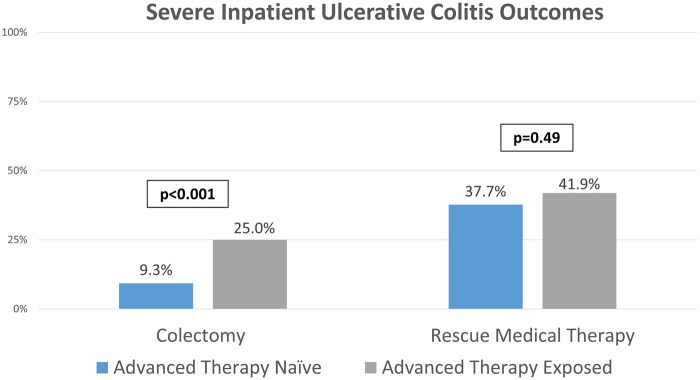
Bar graph demonstrating the rates of colectomy and rescue medical therapy in advanced therapy exposed and naïve cohorts.

In the entire cohort, 47 patients (13%) underwent a colectomy. The rate of colectomy was significantly higher in the ATE group compared to the ATN group (25% vs 9%; *P* < .001). Need for rescue medical therapy did not differ significantly between the ATE and ATN groups, being noted in 41.9% (*n* = 36) in the ATE group vs 37.7% (*n* = 107) in the ATN group (*P* = .49) (Figure 1). The medical rescue therapy agents utilized included infliximab in 131 patients, cyclosporine in 11 patients, and 1 patient received tofacitinib. The median hospital length of stay was 6 days (IQR: 4-9) in both groups (*P* = .96).

### Predictive factors

We wanted to understand what factors predicted the need for colectomy and medical rescue therapy. In particular, we wanted to understand whether prior treatment history (and advanced therapy exposure status) was a key driver of these outcomes, or whether measures of disease severity were better associated with outcomes. Furthermore, we wanted to compare conventional definitions of severity (using the Truelove and Witts criteria) with other measures of disease activity in contemporary practice.

We conducted a series of univariate and multivariate analyses. Factors associated with need for colectomy on univariate analysis included female sex, (61.2% vs 44.8%; *P* = .03), exposure to advanced therapies (44.7% vs 19.9%; *P* < .001), higher baseline median CRP [70.6 mg/dL (IQR: 26.8-126.3) vs 38.8 mg/dL (IQR: 9.7-94.7); *P* = .04], lower baseline albumin [3 g/dL (IQR: 2.7-3.6) vs 3.5 g/dL (IQR: 3-4); *P* < .001], and a Mayo UC endoscopic sub-score of 3 (81.6% vs 55.1%; *P* = .002). Meeting Truelove and Witts criteria was not a significant predictor of colectomy (*P* = .07) (Table S1). On multivariate analysis, baseline albumin level was the only independent predictor of colectomy with an OR of 3.44 [95% CI, 1.63-7.69; *P* = .001] (Table S2). To account for heterogeneity between centers, the treatment center was included as a fixed effect in the multivariate analysis and did not demonstrate any impact on the outcome of the analysis for colectomy.

In patients who required medical rescue therapy, only 41.4% (*n* = 108) met the Truelove and Witts criteria vs 58.6% (*n* = 153) who did not meet the Truelove and Witts criteria (*P* = .06). On univariate analysis, need for medical rescue therapy was associated with baseline CRP (*P* = .05), albumin (*P* = .012), Mayo UC endoscopic sub-score of 3 (*P* = .001), current smoker status (*P* = .015), and CRP/albumin ratio (*P* = .047) (Table S3). Multivariate analysis of predictors for the need of rescue medical therapy is shown in Table S3.

## Discussion

A significant proportion of our current understanding of outcomes after inpatient care of UC comes from studies performed in the pre-biologic era or in predominantly ATN patients. In this multicenter study, we report outcomes among a large contemporary cohort of patients hospitalized for UC and examine the impact of prior advanced therapy exposure as well as markers of disease severity on outcomes. Prior drug exposure is important since it may reflect decreased chance of response to therapy (in line with equivalent observations seen in outpatient clinical trials in moderate/severe UC) and/or it may influence clinician judgment at critical decision-making points such as whether to prescribe medical rescue therapy or recommend colectomy.^10^^,^^13^^,^^16^

We found that the rates of medical rescue therapy did not differ significantly between the ATE and ATN cohorts, but that the colectomy rate was higher in the ATE cohort. Overall lengths of stay were similar in both cohorts. Importantly, advanced therapy exposure status was not a significant predictor of need for either colectomy or medical rescue therapy in multivariate analyses, whereas measures of disease activity, specifically low albumin, were a significant predictor of the need for colectomy. Taken together, we interpret these results to mean that physicians are as likely to offer medical rescue therapy to patients with prior advanced therapy exposure as to more treatment naïve counterparts. However, medical rescue therapy may be less likely to result in benefit in ATE patients. Consequently, patients were more likely to require colectomy in the ATE group. Importantly, our multivariate analysis would suggest that this is driven more by clinician decision-making informed by measures of disease activity such as low albumin, rather than decision-making prejudiced by knowledge of the patient’s treatment history and a consequent sense of “exhaustion” of remaining medical options.

Not all patients in our study met “traditional” Truelove and Witts criteria for acute severe UC. Nevertheless, treating clinicians had decided to admit for inpatient intravenous corticosteroids, and outcomes in terms of colectomy did not differ in the cohort that did not meet traditional criteria. Increasingly, other markers of disease severity are being explored as both markers of severity on admission and potentially predictive of outcomes. For this reason, we explored several other measures of inflammatory burden that correlated with the need for colectomy better than the admission Truelove and Witts criteria. The increasing use of advanced therapies may also be relevant, since administration of these drugs may blunt or alter aspects of the inflammatory or biochemical response. In this regard, it is interesting to note that the CRP:albumin ratio was significantly lower in the ATE group than the ATN group at baseline, despite the ATE cohort subsequently having a significantly higher colectomy rate.

Our study is remarkable for the comparatively high number of patients with prior advanced therapy exposure (*n* = 86). Prior studies include a multicenter retrospective study of 372 patients with ASUC in which only 10% (*n* = 34) had a history of exposure to prior anti-TNF therapy. On multivariate analysis, previous exposure to anti-TNF was significantly associated with the need for colectomy over a 5-year follow-up (OR 3.01, 95% CI, 1.57-5.77, *P* = .001).^14^ However, the authors did not correct for baseline measures of disease activity such as the correction for albumin level, which we find better explains the need for colectomy than prior drug treatment. Indeed, it has been clearly demonstrated that admission albumin level is a major predictor of outcomes in ASUC, including predicting risk of corticosteroid non-response and colectomy.^15^^,^^17^^,^^18^

Another retrospective study of 270 patients with ASUC aimed at developing a colectomy predictive risk score within 1 year included only 34 patients previously exposed to anti-TNF agents and 81 patients exposed to thiopurines.^15^ As in our study, on univariate analysis, anti-TNF exposure was associated with risk of colectomy within 1 year (hazard ratio [HR] 3.17; 95% CI, 1.45-6.93, *P* < .01). On multivariate analysis exposure to anti-TNF or thiopurines (HR 3.86; 95% CI, 1.82-8.18), *C. difficile* infection (HR 3.73; 95% CI, 1.11–12.55), CRP > 30 mg/L (HR 3.06; 95% CI, 1.11-8.43), and albumin < 30 g/L (HR, 2.67; 95% CI, 1.20-5.92) were associated with the risk of colectomy. However, it is unclear if anti-TNF exposure was an independent risk factor for colectomy within 1 year, as the authors grouped exposure to anti-TNF and thiopurines in the multivariate analysis.

Two prior studies are closer in size to our cohort of patients with prior ATE exposure. Sabrie et al reported a single-center retrospective study that aimed at evaluating the outcomes of ASUC patients with outpatient exposure to advanced therapies and included 185 patients (76 previously exposed to advanced therapies).^19^ This study reported no significant difference in the rates of colectomy between the advanced therapies exposed and naïve groups, and the study was limited by a lower number for colectomies to analyze disease severity markers.^19^ On the other hand, Dehghan et al. examined the impact of outpatient biologic therapy exposure in 204 ASUC patients (73 exposed to biologic therapy) and in keeping with the results of our study, found that the colectomy rate within 1 year was significantly higher in the biologic exposed group (52% vs 21%, *P* < .05).^20^ However, no further analysis to control for confounders was performed as this study was published as a conference abstract only.

Our study has multiple strengths. We included patients from multiple centers and 2 different countries, enhancing the generalizability of our results. We included a relatively high number of patients who had been exposed to advanced therapies as outpatients. We were able to control for potential confounders of outcomes by multivariate analyses. Our study is limited by the inherent retrospective design and associated factors such as missing data. We were not able to define the time to colectomy due to missing colectomy dates in some of the patients in our cohort.

In conclusion, outpatient exposure to advanced therapies was not associated with longer hospital stay or higher rates of rescue medical therapy in hospitalized patients receiving intravenous corticosteroid treatment for UC flares. Outpatient exposure to advanced therapies was associated with a higher rate of colectomy compared to those naïve to advanced therapies. However, on multivariate analysis, admission albumin level was the only predictor of colectomy in our cohort. Further larger studies are needed to elucidate the impact of outpatient advanced therapy exposure on outcomes in hospitalized severe UC patients.

## Supplementary Material

otaf055_Supplementary_Data

## Data Availability

The data that support the findings of this study are not publicly available due to privacy, and institutional policies but are available from the corresponding author upon reasonable request.
