# Supplementary material for: Outcomes of Outpatient Advanced Therapy Exposed Patients Hospitalized With Severe Ulcerative Colitis
Source: Crohns Colitis 360. 2025 Aug 12;7(3):otaf055. doi: 10.1093/crocol/otaf055 (PMC12448430; doi:10.1093/crocol/otaf055)
Supplement: otaf055_Supplementary_Data [file otaf055_supplementary_data.zip › Supplementary Tables.docx]

Supplementary Tables

**Supplementary Table 1: Baseline Characteristics Stratified by Colectomy Status**

| Variable | Colectomy  (n=49) | No Colectomy (n=326) | p-value |
| --- | --- | --- | --- |
| Age (years), median (IQR) | 32 (23-49.5) | 35 (25-53) | 0.69 |
| Female, n (%) | 30 (61.2) | 145 (44.8) | **0.03** |
| Disease duration (years), median (IQR) | 2 (1-7) | 2.5 (1-10) | 0.69 |
| Disease location, n (%) |  |  | 0.78 |
| Proctitis | 3 (7.1) | 16 (5.3) |  |
| Left-sided colitis | 16 (38.1) | 105 (34.9) |  |
| Pancolitis | 23 (54.8) | 180 (59.8) |  |
| Smoking status, n (%) |  |  | 0.43 |
| Current smoker | 2 (5.6) | 12 (6.9) |  |
| Previous smoker | 14 (38.9) | 49 (28) |  |
| Never smoker | 20 (55.6) | 114 (65.1) |  |
| Advanced therapy exposed, n (%) | 21 (44.7) | 63 (19.9) | **<0.001** |
| CRP (mg/dL) on admission, median (IQR) | 70.6 (26.8-126.3) | 38.8 (9.7-94.7) | **0.04** |
| Albumin (g/dL) on admission, median (IQR) | 3 (2.7-3.6) | 3.5 (3-4) | **<0.001** |
| CRP/Albumin ratio | 14.6 (5.7-41.9) | 10.7 (2-31) | 0.27 |
| Anemia on admission, n (%) | 20 (41.7) | 114 (35.3) | 0.39 |
| Mayo UC 3 endoscopic sub-score | 31 (81.6) | 141 (55.1) | **0.002** |
| Truelove Witts Criteria, n (%) | 39 (81.3) | 219 (68.2) | 0.07 |
| Concomitant CMV, n (%) | 2 (6.3) | 3 (1.3) | 0.06 |
| Concomitant C. diff, n (%) | 1 (2.1) | 18 (5.9) | 0.28 |
| *IQR: interquartile range; CRP: C-reactive protein; UC: ulcerative colitis; CMV: cytomegalovirus; C. diff: clostridioides difficle* | | | |

**Supplementary Table 2: Univariate and Multivariate Predictors of Colectomy**

| Variable | Univariate analysis | | Multivariate analysis | |
| --- | --- | --- | --- | --- |
|  | **Odds ratio (95% CI)** | **P-value** | **Odds ratio (95% CI)** | **P-value** |
| Age | 0.99 (0.98-1.01) | 0.906 |  |  |
| Female | 1.94 (1.05-3.60) | **0.033** | 1.71 (0.66-4.44) | 0.250 |
| CRP | 1.00 (0.99-1.00) | 0.255 |  |  |
| Albumin | 2.56 (1.56-4.34) | **<0.001** | 3.44 (1.63-7.69) | **0.001** |
| CRP/Albumin Ratio | 1.00 (0.99-1.01) | 0.308 |  |  |
| Mayo UC Endoscopic Sub-score of 3 | 3.61 (1.53-8.50) | **0.003** | 2.36 (0.80-6.92) | 0.116 |
| Anemia | 1.30 (0.70-2.42) | 0.392 |  |  |
| Pancolitis | 0.81 (0.42-1.55) | 0.534 |  |  |
| Disease Duration | 0.97 (0.93-1.01) | 0.229 |  |  |
| Advanced Therapy Exposed | 3.25 (1.72-6.16) | **<0.001** | 1.94 (0.68-5.54) | 0.212 |
| Concomitant C. diff | 0.34 (0.04-2.64) | 0.305 |  |  |
| Concomitant CMV | 5 (0.80-31.14) | 0.085 | 5.29 (0.51-54.51) | 0.161 |
| Current Smoking | 0.58 (0.11-2.91) | 0.512 |  |  |
| EIMs present | 0.54 (0.12-2.42) | 0.422 |  |  |
| *CRP: C-reactive protein; UC: ulcerative colitis; CMV: cytomegalovirus; C. diff: clostridioides difficle. EIMs: extraintestinal manifestations* | | | | |

**Supplementary Table 3: Univariate and Multivariate Predictors of Need for Rescue Medical Therapy**

| Variable | Univariate analysis | | Multivariate analysis | |
| --- | --- | --- | --- | --- |
|  | **Odds ratio (95% CI)** | **P-value** | **Odds ratio (95% CI)** | **P-value** |
| Age | 0.99 (0.98-1.00) | 0.601 |  |  |
| CRP (mg/dL) on presentation (U) | 1.00 (1.00-1.00) | **0.050** | 0.98 (0.95-1.00) | 0.092 |
| Sex |  |  |  |  |
| Male |  |  |  |  |
| Female | 0.74 (0.49-1.13) | 0.172 |  |  |
| Concomitant CMV |  |  |  |  |
| Yes | 2.36 (0.38-14.37) | 0.351 |  |  |
| No |  |  |  |  |
| Albumin (g/dL) on presentation | 1.54 (1.10- 2.15) | **0.012** | 0.68 (0.32-1.43) | 0.320 |
| Flexible sigmoidoscopy |  |  |  |  |
| Mayo 1+2 |  |  |  |  |
| Mayo 3 | 2.26 (1.39-3.67) | **0.001** | 1.99 (0.77-5.14) | 0.155 |
| Hemoglobin |  |  |  |  |
| < 11.5 g/dL | 0.90 (0.58-1.39) | 0.647 |  |  |
| ≥ 11.5g/dL |  |  |  |  |
| Pancolitis |  |  |  |  |
| Yes | 0.99 (0.64-1.54) | 0.982 |  |  |
| No |  |  |  |  |
| Smoking |  |  |  |  |
| Current | 0.14 (0.02-0.68) | **0.015** | 0.42 (0.06-2.60) | 0.352 |
| Previous |  |  |  |  |
| Disease Duration | 0.98 (0.96-1.01) | 0.389 |  |  |
| Advanced Therapy Status |  |  |  |  |
| Naïve |  |  |  |  |
| Exposed | 1.19 (0.72-1.94) | 0.485 |  |  |
| CRP/Albumin Ratio | 1.00 (1.00-1.01) | **0.047** | 1.06 (0.99-1.14) | 0.062 |
| EIM Present |  |  |  |  |
| Yes | 1.25 (0.51-3.02) | 0.614 |  |  |
| No |  |  |  |  |
| Previous anti-TNF |  |  |  |  |
| Yes | 1.15 (0.69-1.92) | 0.579 |  |  |
| No |  |  |  |  |
| Concomitant C. diff |  |  |  |  |
| Yes | 0.43 (0.14-1.33) | **0.145** | 1.18 (0.21-6.56) | 0.849 |
| No |  |  |  |  |
| *CRP: C-reactive protein; UC: ulcerative colitis; CMV: cytomegalovirus; C. diff: clostridioides difficle. EIMs: extraintestinal manifestations. TNF: tumor necrosis factor.* | | | | |
